# Whole-Genome Sequence of an Indian Group A *Streptococcus emm* Type 1-2 Strain Isolated from a Blood Sample in North India

**DOI:** 10.1128/MRA.00163-20

**Published:** 2020-05-07

**Authors:** Vivek Sagar, Anuradha Chakraborti, Rajesh Kumar

**Affiliations:** a Community Medicine & School of Public Health, Post Graduate Institute of Medical Education & Research (PGIMER), Chandigarh, India; b Experimental Medicine & Biotechnology, Post Graduate Institute of Medical Education & Research (PGIMER), Chandigarh, India; University of Maryland School of Medicine

## Abstract

Group A *Streptococcus* *emm* type 1-2 is more prevalent than *emm* type 1 in India. Only partial information is available about the genetic characteristics of this type. Here, genome sequencing of *emm* type 1-2 strain 1085 (from blood) was conducted. A contig 2,010,300 bp long, with a total of 1,877 annotated proteins, was obtained (NCBI accession number CP047120, assembly accession number ASM983284v1).

## ANNOUNCEMENT

Group A *Streptococcus* (GAS) (Streptococcus pyogenes), which is responsible for many life-threatening invasive infections, is a highly diverse and rapidly emerging pathogen (1, 2). As more than 200 *emm* types of GAS have been identified worldwide, attempts to develop a vaccine have failed (3). Even *emm*-typing data from India are different from those of other countries (3–6). Our previous data showed that *emm*1, which is prevalent worldwide, is rare in India. Instead, *emm*1-2 strains are more common in India (7). Type *emm*1-2 strains have been isolated from throat, skin, and also blood samples, indicating its invasive nature. Our reports based on PCR and microarray analysis have explored little about this type (5, 7). So, whole-genome sequencing of a type *emm*1-2 strain was carried out here.

Our study was approved by the Institute Ethics Committee (IEC) of Post Graduate Institute of Medical Education & Research (PGIMER) in Chandigarh, India (INT/IEC/2018/000760). The *emm*1-2 strain (1085) was isolated from a blood sample of a patient suffering from septicemia who was hospitalized at PGIMER (3, 8). The strain was isolated using a blood culturing technique as per the standardized protocol. For genomic DNA isolation, Todd-Hewitt broth with 0.2% yeast extract was used. Culturing was done at 37°C. Genomic DNA was isolated using zirconium beads in combination with a DNeasy kit (Qiagen, Germany). A hybrid assembly was generated from Illumina (Genome Analyzer IIx; RTA version 1.8.70.0) and Nanopore (GridION-X5) data. Libraries of 320 bp were prepared according to Illumina’s instructions, “Preparing Samples for Paired-End-Sequencing.” For Nanopore data, DNA from the sample was end repaired using the NEBNext Ultra II end repair kit (catalog number E7546L; New England BioLabs, USA) and cleaned up with 1× AmPure beads (Beckmann Coulter, USA). Native barcode ligation was performed with a blunt/TA (T4 DNA) ligase mix (M0367L; New England BioLabs) using the native barcoding genomic DNA kit (EXP-NBD104; Oxford Nanopore Technologies, UK) and cleaned with 1× AmPure beads. Qubit-quantified barcode-ligated DNA sample libraries were pooled at equimolar concentrations to attain a 600-ng pooled sample. Adapter ligation (BAM) was performed for 10 min using the NEBNext quick ligation module (E6056L; New England BioLabs). The library mix was cleaned up using 0.6× AmPure beads, and finally, the sequencing library was eluted in 15 μl of elution buffer. Sequencing was performed on a SpotON flow cell R9.4 (Oxford Nanopore Technologies). For quality checking, the minimum threshold quality score for Nanopore data was a Q-score of 7. Raw data were also error corrected using Illumina data and assembled using Maryland Super Read Cabog Assembler (MaSuRCA) version 3.3 (9). The gene and protein predictions were carried out using Prokka version 1.14 (10). The predicted proteins were used for a similarity search using the Diamond version 0.8.29 BLASTP program (11). All software was used with default parameters.

**FIG 1:**
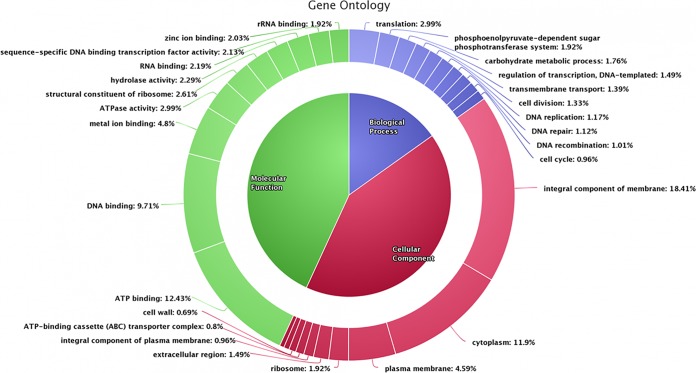
Gene ontology (GO) association of predicted protein-coding genes of the *emm*1-2 GAS strain 1085.

Approximately 194,811 reads were generated. The number of reads by Illumina sequencing was 10,184,236, with an absolute length of 2 × 110 bp. For Nanopore sequencing, the total read length was 350,972,921, with an average of 1,801.6 bp and an *N*_50_ value of 2,807 bp. The assembly resulted in a contig 2,010,300 bp long, which showed homology with GAS. The G+C content of 38.4% was comparable with that in earlier reports (12). Out of a total of 2,055 genes, 1,937 were complementary determining sequences (CDSs) and 1,877 were annotated (Fig. 1). A total of 118 genes coding for different RNAs were also identified, of which 8 each encoded 5S, 16S, and 23S rRNAs.

### Data availability.

This information has been deposited at DDBJ/EMBL/GenBank under the accession number CP047120. The raw data were submitted to the Sequence Read Archive (SRA) with the accession number PRJNA596618.

## References

[1] Walker MJ, Barnett TC, McArthur JD, Cole JN, Gillen CM, Henningham A, Sriprakash KS, Sanderson-Smith ML, Nizet V 2014 Disease manifestations and pathogenic mechanisms of group A *Streptococcus*. Clin Microbiol Rev 27:264–301. doi:10.1128/CMR.00101-13. 24696436 PMC3993104

[2] Carapetis JR, Steer AC, Mulholland EK, Weber M 2005 The global burden of group A streptococcal diseases. Lancet Infect Dis 5:685–694. doi:10.1016/S1473-3099(05)70267-X. 16253886

[3] Sagar V, Bergmann R, Nerlich A, McMillan DJ, Nitsche P, Chhatwal GS 2012 Variability in the distribution of genes encoding virulence factors and putative extracellular proteins of *Streptococcus pyogenes* in India, a region with high streptococcal disease burden, and implication of development of a regional multi-subunit vaccine. Clin Vaccine Immunol 19:1818–1825. doi:10.1128/CVI.00112-12. 22971782 PMC3491543

[4] Sagar V, Bakshi DK, Nandi S, Ganguly NK, Kumar R, Chakraborti A 2004 Molecular heterogeneity among North Indian isolates of group A *Streptococcus*. Lett Appl Microbiol 39:84–88. doi:10.1111/j.1472-765X.2004.01545.x. 15189292

[5] Sagar V, Kumar R, Ganguly NK, Chakraborti A 2008 Comparative analysis of emm type pattern of group A *Streptococcus* throat and skin isolates from India and their association with closely related SIC, a streptococcal virulence factor. BMC Microbiol 8:150. doi:10.1186/1471-2180-8-150. 18796133 PMC2556678

[6] Dhanda V, Kumar R, Thakur JS, Chakraborti A 2010 emm type distribution pattern of group A *Streptococcus* in North India: need for a new preventive approach. Ind J Med Res 132:741–744. PMC3102467 21245627

[7] Sagar V, Nerlich A, Bergmann R, Nitsche P, McMillan DJ, Geffers R, Hoe N, Kumar R, Nitsche P, Fulde M, Talay S, Rohde M, Chakraborti A, Chhatwal GS 2014 Differences in virulence repertoire and cell invasive potential of group A *Streptococcus* emm1-2 in comparison to emm1 genotype. Int J Med Microbiol 304:685–695. doi:10.1016/j.ijmm.2014.04.011. 24856243

[8] Haggar A, Nerlich A, Kumar R, Abraham VJ, Brahmadathan KN, Ray P, Dhanda V, Joshua JMJ, Mehra N, Bergmann R, Chhatwal GS, Norrby-Teglund A 2012 Clinical and microbiologic characteristics of invasive *Streptococcus pyogenes* infections in North and South India. J Clin Microbiol 50:1626–1631. doi:10.1128/JCM.06697-11. 22357508 PMC3347136

[9] Zimin AV, Marçais G, Puiu D, Roberts M, Salzberg SL, Yorke J 2013 The MaSuRCA genome assembler. Bioinformatics 29:2669–2677. doi:10.1093/bioinformatics/btt476. 23990416 PMC3799473

[10] Seemann T 2014 Prokka: rapid prokaryotic genome annotation. Bioinformatics 30:2068–2069. doi:10.1093/bioinformatics/btu153. 24642063

[11] Buchfink B, Xie C, Huson DH 2015 Fast and sensitive protein alignment using DIAMOND. Nat Methods 12:59–60. doi:10.1038/nmeth.3176. 25402007

[12] McShan WM, Ferretti JJ, Karasawa T, Suvorov AN, Lin S, Qin B, Jia H, Kenton S, Najar F, Wu H, Scott J, Roe BA, Savic DJ 2008 Genome sequence of a nephritogenic and highly transformable M49 strain of Streptococcus pyogenes. J Bacteriol 190:7773–7785. doi:10.1128/JB.00672-08. 18820018 PMC2583620

